# Sensory-Motor Polyneuropathy and Digital Ischemia: A Rare Presentation of Granulomatosis with Polyangiitis

**DOI:** 10.1155/2021/5353575

**Published:** 2021-10-30

**Authors:** Wasundara Wathurapatha, B. G. A. Rathnamali, Upul Dissanayake

**Affiliations:** National Hospital of Sri Lanka, Colombo, Sri Lanka

## Abstract

Granulomatosis with polyangiitis (GPA) typically presents with upper or lower respiratory tract symptoms and/or with renal involvement. Although it can affect the peripheral nervous system frequently, with mononeuritis multiplex being the most common pattern, the occurrence of peripheral sensory-motor polyneuropathy as a presenting manifestation is distinctly rare. Prevalence of digital gangrene is also extremely rare in GPA. We describe a 46-year-old woman presenting with severe peripheral sensorimotor polyneuropathy affecting bilateral lower limbs preceded by a purpuric skin rash and multiple painful ulcers confined to the lower limbs. She had evidence of digital ischemia affecting multiple toes and dry gangrene of the left 4^th^ toe. Diagnosis of GPA was made based on skin biopsy, positive ANCA serology, and clinical criteria. She made a good recovery following aggressive immunosuppressive treatment with methylprednisolone and cyclophosphamide and was maintained on prednisolone and azathioprine. This case highlights the importance of suspecting GPA in a patient presenting with sensorimotor polyneuropathy and/or digital ischemia even in the absence of more classic presenting features and underlies the necessity of accurate differential diagnosis in evaluating a case of peripheral neuropathy.

## 1. Introduction

ANCA-associated vasculitis (AAV) is the commonest primary systemic small-vessel vasculitis (SVV) in adults which consists of granulomatosis with polyangiitis (GPA), eosinophilic granulomatosis with polyangiitis (EGPA), and microscopic polyangiitis (MPA). GPA is characterized by the presence of necrotizing granulomatous inflammation in the absence of asthma and eosinophilia [1]. GPA is a relatively rare disease with a prevalence of 24–152 cases per million [2]. If untreated, the disease usually runs a rapidly fatal course, and 82% of patients die within 1 year [3]. Thus, accurate and prompt diagnosis of GPA is of utmost importance to improve the prognosis.

It mostly affects the upper or lower respiratory tracts and kidneys causing otorhinolaryngological manifestations and crescentic glomerulonephritis [2]. Although significant proportion of patients with GPA develop peripheral nervous system (PNS) involvement during disease course, presentation with peripheral neuropathy is rare, as discussed in the following. Furthermore, according to the published literature, the prevalence of digital ischemia and gangrene in the GPA population is extremely rare as ≤1% [4, 5]. We report a case of GPA presenting with distal sensory-motor polyneuropathy and digital ischemia both of which are atypical and rare presenting features of GPA. The steps in achieving the diagnosis of GPA including the diagnostic criteria are discussed as well as the treatment regimen.

## 2. Case Report

A 46-year-old woman presented with progressive bilateral lower limb weakness for 2 weeks. This was associated with burning paresthesia and numbness up to the knees. Five days prior to the onset of weakness, she has noticed a skin rash over her soles and multiple painful ulcers over the bilateral lower limb. Concurrently, she has had pain and bluish discoloration of 2nd–5th toes bilaterally which soon evolved into gangrene in the left 4th toe. She had no upper limb, neck, bulbar, or respiratory muscle weakness nor features of dysautonomia. She did not have fever, joint pains, oral ulcers, genital ulcers, or photosensitivity rashes. There was no history of Raynaud phenomenon, hemoptysis, or hematuria. Over the last three years, she has taken medical opinion several times for nasal congestion, symptoms of upper respiratory infections, sinusitis, and one episode of epistaxis. Otherwise, her medical history was insignificant. She was not on any chronic medication. She does not smoke and does not consume alcohol or any illicit substances.

**Figure 1:**
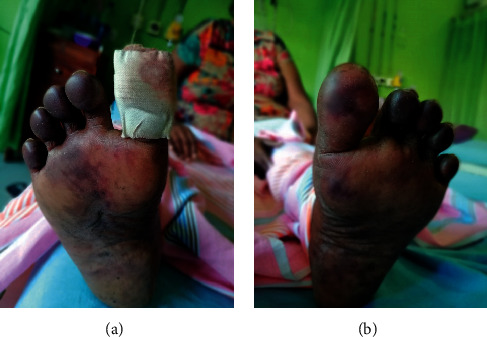
Patient's feet showing retiform purpura and evidence of digital ischemia.

**Figure 2:**
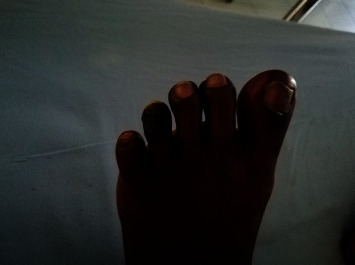
Dry gangrene of the left 4th toe.

**Figure 3:**
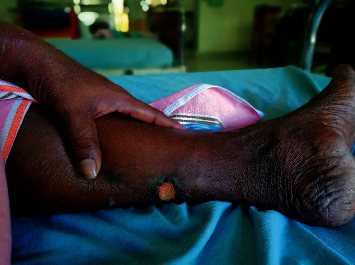
Punched out ulcer over the left calf.

On examination, there were palpable retiform purpuric patches over the plantar surfaces of her feet together with bluish/black discoloration of bilateral 2nd–5th toes (Figure 1) and dry gangrene of the left 4th toe (Figure 2). Few punched out ulcers were present over both legs below the knees (Figure 3). Both posterior tibial and dorsalis pedis pulses were present bilaterally. Skin manifestations were confined to the lower limbs, and there was no involvement of mucosal membranes. She did not have other features suggestive of connective tissue disorders such as malar rash, oral ulcers, photosensitivity, or thickened skin. There was no evidence of active synovitis or deforming arthritis.

Neurological examination revealed bilateral flaccid foot drop, distal motor weakness with grade 2 power, and absent knee and ankle jerks bilaterally. All modalities of sensation were reduced up to the knee in the left leg and up to the midcalf in the right leg. Her upper limbs were neurologically normal, and there was no bladder bowel involvement, postural hypotension, or respiratory compromise. She did not have cerebellar signs, and her higher functions were normal.

Respiratory examination revealed a respiratory rate of 13 breaths per minute, midline trachea, and vesicular breathing with no added sounds. Her heart rate was 80 beats per minute and regular, blood pressure was 140/80 mmHg, and there were no cardiac murmurs. The rest of her examination was unremarkable.

Investigations showed an erythrocyte sedimentation rate (ESR) of 110 mm in the first hour, positive cytoplasmic antineutrophil cytoplasmic antibody (c-ANCA), and associated proteinase 3 ANCA (PR3-ANCA). However, she was negative for perinuclear ANCA (p-ANCA), antinuclear antibody (ANA), rheumatoid factor, and multiple other autoantibodies.

Nerve conduction study (NCS) showed severe sensorimotor axonal polyneuropathy (asymmetrical), and the skin biopsy revealed leukocytoclastic vasculitis with fibrinoid necrosis of vessel walls. There were changes of sinusitis in both maxillary sinuses (Right > left) as shown in her noncontrast CT paranasal sinuses, but no nasal polyps or bone destruction. Nasal septal biopsy report described nonspecific chronic inflammatory changes, but no granulomas were noted. A summary of the patient's investigations is given in Table 1.

Upon strong clinical suspicion of vasculitis, she was immediately started on intravenous (IV) methylprednisolone pulse 1 g/day for 3 days followed by oral prednisolone 60 mg daily, which was slowly tapered over the next several months. A diagnosis of GPA was made based on the clinical picture and the results of ANCA testing and skin biopsy. Thereafter, she was given 6 pulses of IV cyclophosphamide 500 mg, initial 3 pulses at 2-week intervals and then every three weeks. Physiotherapy was commenced. She responded well to therapy with the improvement of skin manifestations, excruciating lower limb pain, reversible tissue ischemia, and cessation of further extension of digital gangrene. Her lower limb power gradually improved to grade 4. At follow up, after completion of cyclophosphamide pulses, she has made a good recovery and was able to walk with support. She was subsequently followed-up at the rheumatology clinic and was maintained on azathioprine 50 mg and tapering dose regimen of prednisolone.

## 3. Discussion

Granulomatosis with polyangiitis (GPA) is a type of ANCA-associated SVV that preferentially involves venules, capillaries, and arterioles. However, in spite of this preferential involvement, SVV can affect medium or even large-sized arteries overlapping with the clinical spectrum of medium and large-vessel vasculitis [1]. The pathological hallmark of GPA is necrotizing granulomatous inflammation that affects vasculature or appears solely in extravascular tissue [6]. Although GPA characteristically involves with upper or lower respiratory tract or kidneys with a prevalence of 90% and 78%, respectively [2, 7], a wide spectrum of manifestations may be evident at the initial presentation and throughout the disease course. Our patient presented with painful, distal, asymmetrical sensory-motor axonal polyneuropathy and cutaneous manifestations including digital gangrene without renal or lower respiratory tract involvement. This is an atypical presentation of GPA.

Prevalence of PNS involvement in GPA has been reported as 10.6–43.8% [8–10]. However, peripheral neuropathy occurring as a presenting manifestation of GPA is as rare as 2.4–8% [10, 11]. Among those with PNS involvement, mononeuritis multiplex was the commonest pattern, whereas distal sensory-motor polyneuropathy was uncommon [10]. Although peripheral neuropathy is usually not the cause of death in AAV, it is associated with significant pain and disability, impairing the quality of life [7, 12]. The pathophysiology of vasculitic neuropathy (VN) is inflammation of vasa nervorum followed by thrombosis and occlusion leading to ischemia of the peripheral nerve with axonal degeneration [13]. According to the peripheral nerve society guidelines, typical features of VN are sensory or sensory-motor involvement (not pure motor), multifocal or asymmetric distribution, predilection for lower limbs, distal predominance, pain, and acute relapsing course (onset to maximum severity within one month followed by spontaneous stabilization or improvement of neurological deficits). NCS in VN characteristically demonstrate asymmetric or nonlength-dependent patterns of axonal neuropathy [14]. Therefore, our patient's clinical and NCS findings were compatible with VN. There were no features suggestive of alternative diagnoses such as nonvasculitic paraneoplastic neuropathy, hereditary neuropathy, amyloidosis, and sarcoidosis which can cause a similar pattern of neuropathy.

Histopathological examination of nerve biopsy is the gold standard investigation for diagnosis of VN [14]. As a limitation, we were not able to perform a nerve biopsy to achieve a definite diagnosis of VN. However, one previous study has shown that only 12% of patients with ANCA-associated vasculitis (AAV) have undergone nerve biopsy, and majority of patients (56%) were categorized as having “possible VN” without performing a biopsy. The diagnosis of VN is not routinely confirmed by nerve biopsy in usual clinical practice, but it is rather supported by the presence of active systemic vasculitis [8]. The Brighton Collaboration Vasculitic Peripheral Neuropathy Working Group has recommended a case definition of VN with varying degrees of diagnostic certainty [15]. We made a diagnosis of probable VN based on this case definition as her skin biopsy confirmed the diagnosis of SVV. Among patients with AAV, those with VN more often had skin, musculoskeletal, and cardiovascular involvement. In contrast, they were reported as having less renal and pulmonary involvement or life-threatening manifestations of vasculitis [8, 11]. Similarly, our patient had associated cutaneous manifestations but not renal/pulmonary involvement.

Cutaneous manifestations have been reported in 14–50% of patients with GPA, with palpable purpura being the most common feature [5, 16]. Again, a greater predilection for lower limbs has been observed [16]. Less common features were skin ulcers, skin nodules, necrotic papules, pustules, oral ulcers, gingival hyperplasia, palpebral xanthoma, genital ulcers, livedo reticularis, and digital necrosis. The prevalence of digital necrosis among 75 cases of GPA was as low as 1.3% [5]. Similarly, another review revealed that prevalence of digital ischemia and gangrene in the GPA population is likely to be <1%, given the paucity of reported cases [4]. In GPA, digital gangrene can occur at initial presentation or develop later in the course of the disease, even while on active treatment [4]. The most commonly observed histopathological pattern of cutaneous GPA was leukocytoclastic vasculitis [16]. The immediate cause of digital ischemia and gangrene is most probably the destruction of medium-sized vessels due to active vasculitis as suggested by histopathological examination of affected tissues [17, 18]. However, it can be due to in situ thrombosis as a result of active vasculitis as seen in angiographic examination of affected patients as well as on histological examination [19, 20]. Histological examination of toe gangrene in our patient revealed vasculitis with no evidence of thromboembolic phenomenon. One limitation was that we were unable to perform an angiogram of her lower limbs. Since the peripheral pulses of her feet were palpable, angiogram was not recommended by the vascular surgical team. In contrast, peripheral pulses were absent in previously reported cases in which angiograms were performed [19, 20].

Our patient had positive c-ANCA detected by indirect immunofluorescence (IIF) and PR3-ANCA detected by enzyme immunoassays (ELISA). Combining IIF and ELISA enhances the sensitivity and specificity of a diagnosis of AAV to 96% and 98.5%, respectively [21]. However, the gold standard for diagnosing AAV is histopathological evidence of necrotizing vasculitis in any organ [22]. Therefore, our patient was diagnosed as having AAV based on positive c-ANCA and histopathology of skin biopsy. EGPA can be excluded due to absence of asthma and eosinophilia in our patient. GPA is differentiated from MPA by the presence of granulomatous inflammation on biopsy [1]. In our patient, skin biopsy did not reveal any granulomas, and the nasal septal biopsy was nonspecific. Often, the definitive granulomas are missed in the biopsies due to the sampling error, which makes differentiating the type of AAV difficult [4]. Literature has shown that 90% of nasal systematic biopsies performed under local anesthesia were nonspecific [23]. The presence of c-ANCA/PR3-ANCA is highly specific for GPA, and it is the typical ANCA pattern that occurs in GPA [24]. According to 2017 ACR/EULAR provisional classification criteria for GPA, a combined score of ≥5 is needed for classification of GPA with a sensitivity of 91% and specificity of 94% [25]. Our patient scores 3 points on clinical criteria given her history of nasal congestion, sinusitis, and epistaxis and scores 5 points on serologic criteria due to positive c-ANCA/PR3 antibody. Therefore, she can be classified as having GPA based on the total score of 8.

The treatment of AAV consists of remission induction and maintenance of remission. Glucocorticoids and either cyclophosphamide or rituximab combination is the recommended treatment for remission induction in the setting of new onset organ or life-threatening AAV. Because of high cost, rituximab is restricted for specific circumstances where rituximab is preferred over cyclophosphamide, e.g., for patients who are in their reproductive age. For remission maintenance, low-dose glucocorticoids in combination with either azathioprine, methotrexate, mycophenolate mofetil, or rituximab are recommended with azathioprine being the most preferred. Following induction of remission, maintenance therapy should be continued for at least 24 months [22]. Neuropathies associated with systemic vasculitis should be treated according to the guidelines of the underlying disease [26]. A study involving 40 patients with AAV complicated with VN revealed that all patients who received this aggressive immunosuppressive therapy attained remission of neuropathy within 9 months and 35% of them did not have any long-term sequelae of peripheral neuropathy [11]. As documented in the literature, glucocorticoids and cyclophosphamides are the most commonly used treatment for digital ischemia and gangrene [4]. Our patient was treated with glucocorticoids and cyclophosphamide pulse therapy to achieve remission and subsequently maintained on GC and azathioprine. She made a good recovery with minimal residual disability from peripheral neuropathy, and now, she has remained in remission for more than 1 year.

In conclusion, peripheral sensorimotor polyneuropathy can be a presenting manifestation of systemic SVV. Therefore, patients with peripheral neuropathy of an unclear origin should be investigated thoroughly and closely followed-up to detect a possible underlying GPA. Furthermore, digital ischemia can also be a rare manifestation of GPA despite being a SVV. As both of these conditions warrant aggressive immunosuppressive treatment, early diagnosis is important. Maintaining a high index of suspicion for GPA is important for the physicians to diagnose GPA early, which significantly reduces the morbidity and mortality of this fatal disease.

## Figures and Tables

**Table 1:** Summary of investigations.

Investigation	Value	Reference range
White cell count	9300	4,000–11,000 cells/*μ*L
Neutrophils	6600	2,000–7,000 cells/*μ*L
Lymphocytes	1120	1,000–4,000 cells/*μ*L
Hemoglobin	12.0	12–16 g/dL
Platelets	177,000	150,000–450,000/*μ*L
Blood film	No abnormal cells seen. Within normal limits	
Serum creatinine	65	70–110 *μ*mol/L
Sodium	136	135–145 mmol/L
Potassium	4.2	3.5–4.5 mmol/L
AST	20	<40 U/L
ALT	25	<35 U/L
Albumin	35	35–45 g/L
Globulin	38	30–40 g/L
ALP	106	30–120 U/L
ESR	110	<20 mm/1st hour
CRP	36	<6 mg/L
Rheumatoid factor	<8	<8 IU/L
Antinuclear antibodies	Negative	
HIV 1 and 2 antigens/antibodies	Negative	
c-ANCA	Positive	
p-ANCA	Negative	
Anti-Smith antibody	Negative	
Anti-Scl-70 antibody	Negative	
Anti-Ro antibody	Negative	
Anti-La antibody	Negative	
Lupus anticoagulant	Negative	
Anticardiolipin antibody, IgM	Negative	
Anti-*ß*_2_ glycoprotein antibodies, IgM and IgG	Negative	
Serum complement level	Normal	
Serum cryoglobulin	Negative	
Hepatitis C antibody	Negative	
Hepatitis B surface antigen	Negative	
Urine full report	Normal	
Chest X-ray	No abnormality detected	
Ultrasound abdomen	Normal	
2D echocardiogram	No abnormality detected including any cardiac source of emboli	
HbA1C	6.0%	

AST, aspartate transaminase; ALT, alanine transaminase; ALP, alkaline phosphatase; ESR, erythrocyte sedimentation rate; CRP, C-reactive protein; TSH, thyroid stimulating hormone; HIV, human immunodeficiency virus; c-ANCA, cytoplasmic antineutrophil cytoplasmic antibody; p-ANCA, perinuclear antineutrophil cytoplasmic antibody.
